# The Physiological and Therapeutic Action of Acetanilide or Antifebrin

**Published:** 1887-04

**Authors:** F. R. Campbell


### Translations.

### *THE PHYSIOLOGICAL AND THERAPEUTIC ACTION OF ACETANILIDE OR ANTIFEBRIN.*

By DR. WEILL.

Translated from *Bull. Gen. de Therapeutique*, by F. R. CAMPBELL, M. D.

At the request of our esteemed chief, Prof. Dujardin-Beaumetz, we have undertaken an experimental study of a new remedy, acetanilide, and now communicate the results of our investigation.

*History.*—Acetanilide has long been known to chemists, but it is only within a few months that it has attracted the attention of the medical world. To Drs. A. Cahn and P. Hepp, assistants at the clinic of Kussmaul of Strasbourg, is due the honor of having for the first time studied the therapeutic action of this substance.* These gentlemen have employed acetanilide in a number of fevers, in doses of from four to fifteen grains, the dose never exceeding thirty grains. A dose of four grains of acetanilide is equal to a dose of fifteen grains of antipyrine, and to produce a rapid and energetic effect it is better to give a single large dose than a number of small ones.

"None of our patients," say Cahn and Hepp, "have complained of the medicine, and their general condition during the periods of apyrexia was excellent. The only troublesome symptom was a slight cyanosis of the face and extremities, but this phenomenon rapidly disappears."

Shortly after the publication of the article of Cahn and Hepp, M. Lepine of Lyons gave the results of his physiological and clinical experiments.† This eminent professor after having studied the

**Centralblatt fuer klinische Med.*, Sept., 1886.

† *Lyons Medical*, 1886, No. 44.

action of the drug upon the temperature, circulation, blood and nervous system of animals, employed it in non-febrile diseases, especially in locomotor ataxia for the lightning pains. Lepine concludes that acetanilide is not only an antithermic but also possesses valuable properties as a nerve tonic and sedative.

M. Mouisset, Prof. Lepine's assistant, published his observations on the use of antifebrin in seven cases of typhoid fever. Mouisset found that it was necessary to give seven and one half grains of acetanilide to obtain the effect produced by sixteen grains of antipyrine. The drug is more active if given but once or twice in twenty-four hours, as the system becomes accustomed to its presence if given more frequently. Cyanosis was observed three times, but it was never accompanied by *malaise* and was always of a very transitory character.

When Lepine's article was published, Krieger gave a report of his experience with the drug in eighteen cases of fever (four typhoid, three malarial, four pneumonia, four rheumatism, two pleurisy, one diphtheria, one puerperal.) According to him acetanilide was much more efficacious as an antithermic in typhoid and malarial fevers than in other acute diseases. Krieger has also used acetanilide successfully in the treatment of facial neuralgia, and claims that it has antiseptic properties.

*Chemistry of Acetanilide.*—Acetanilide is a white, odorless, and almost tasteless crystalline substance. It is soluble in 160 parts of cold water and in 50 parts of warm water; in three and one-half parts of alcohol, six of ether and seven of chloroform.

It melts at 105° C. and volatilizes at 292° C. When heated it is decomposed by the action of acids and alkalis forming aniline and acetic acid. The formula of acetanilide is $C^8 H^9 N$, and was discovered in 1845 by Gerhardt. It has also been called *phenylacetamide.*

*Pharmacology.*—Acetanilide may be prepared in many ways. One of the most convenient is the following: In a one-litre flask placed in a sand bath and connected with a condenser surrounded by cold water, place pure colorless aniline, 372 grammes; crystallizable acetic acid, 240 grammes. Heat to the boiling point and maintain the temperature for four hours. The vapors condensed in the still should flow back into the flask. The acetanilide thus formed is separated by successive crystallizations. The test for acetanilide is to agitate the suspected substance with ether, decant and evaporate to dryness, and pour upon the residue some drops of pure sulphuric acid with a crystal of bichromate of potash. If acetanilide is present a characteristic rose-colored precipitate is formed.

Another test is to agitate the suspected liquid with chloroform, decant and evaporate the chloroform in a capsule containing a little protonitrate of mercury, when a green color appears.

With these delicate tests the urine of patients who had taken from twenty to thirty grains of acetanilide a day was many times tested and the substance was never detected. But when some crystals of acetanilide were dissolved in a litre of normal urine the characteristic re-actions were always observed. We may then conclude that the drug is not eliminated by the kidneys as acetanilide.

Acetanilide should not be used,

1. If it is not odorless.

2. If it is not white.

3. If it is not converted into a colorless liquid when heated upon platinum foil.

4. If it is not entirely volatile.

5. If it gives with hypobromite of soda a red orange precipitate. This last re-action, which is very delicate, takes place whenever there are traces of anilide in the anti-febrin.

*Posology.*—Acetanilide may be given in capsules or dissolved in a little wine. The dose varies from four to eight grains, according to the disease and the effect desired. The dose should not exceed eight grains whatever the quantity given in 24 hours.

PHYSIOLOGICAL STUDY OF ACETANILIDE.

§ 1. *General Physiological Effects.*—In small doses, acetanilide has no effect upon man or animals in a normal state. Injected subcutaneously in rabbits and Guinea pigs, or inserted through an œsophageal tube into the stomach of dogs, the drug produces no disturbance, no lowering of temperature. Four hours after breakfast I took a dose of one drachm of acetanilide in wine without observing any effect upon the physiological functions whatever. The temperature taken every fifteen minutes, remained normal, the smygmographic tracing of the pulse was unchanged, and there was no peculiar sensation which attracted our attention.

In larger doses, two to four grains to the pound of the animal, acetanilide is poisonous and we observe a train of symptoms which terminate in death. Within a few minutes following the ingestion of the drug the animal manifests symptoms of weakness, stupor, slowness of movement and a rapid and steady reduction of temperature. Although the animal was wrapped in flannel, the re-action was but slight, the respiration was slowed and then became irregular; soon the body becomes cold. There is anæsthesia of the posterior part of the body; sensation is at first diminished and then disappears completely, although the anterior part of the body is still susceptible to pain. Lastly collapse comes on attended with convulsive movements. These convulsions are most marked in the rabbit, occurring two or three times a second and continuing until death. Death comes on slowly, occurring usually within from twenty-four to thirty-six hours after the ingestion of the drug.

§ 2. *Action of Acetanilide upon the Principal Physiological Functions.*—In toxic doses respiration is retarded, becomes deep, and a tendency to asphyxia is observed. The action of the heart is at first accelerated and the impulse increased; later the pulse becomes slow and irregular. The same increase of the systolic impulse in the first period is observed in the frog. The blood pressure is also modified. The first effect upon the circulatory system is to increase the amplitude of the intravascular oscillations. Then the smygmographic tracings show a decrease of blood pressure and the pulse becomes frequent and irregular.

In a rabbit to which eight grains of acetanilide had been administered subcutaneously, we observed that the blood vessels of the ear, dilated before the experiment, soon became contracted, anæmic and cold.

The elements of the blood undergo important changes. The quantity of oxyhæmoglobin in a dog weighing seventeen pounds, to which eighty grains of acetanilide had been administered, fell gradually from 12 to 5½ per cent. Two hours after the ingestion of the drug the spectroscopic band showing the presence of methæmoglobin appeared.

We have already stated that smaller doses of acetanilide have no effect upon the temperature, but in large doses a very marked antithermic effect is produced, the temperature falling eight or ten degrees below normal. This reduction of temperature is at first peripheral, but soon becomes central. It begins within a few minutes after the administration of the drug, and attains its maximum in about four hours. In a rabbit a dose of eight grains reduced the temperature 1½° C. A dose of twenty-two grains reduced the temperature 8° C. The animal to which this last dose had been given lived twenty-four hours with temperature reduced, before dying.

*Action on the Nervous System.*—Acetanilide has an undoubted action on the nervous system, as is clearly shown by the results of chemical and experimental studies. It is the experimental study which has given valuable information regarding its action in this way.

*a.* Acetanilide in small doses seems to have no effect upon the functions of the cerebrum. The animals retain all their intelligence, respond to caresses, and remain in possession of their will power. It is only in the last stage of the intoxication when the phenomena of asphyxia have appeared that the cerebral functions seem to be abolished with those of the nervous system in general.

*b.* But the functions of the spinal cord and medulla are constantly affected when the drug is given in sufficient doses. Reflex action is diminished in a marked degree, and, as we have observed before, the excitability of the pneumogastric nerve is perceptibly lowered, although it is not abolished. The action of the drug upon the bulbo-spinal nervous system seems to be manifested by a reduction of the excito-motor functions, as is shown by the phenomena of collapse, loss of motion, and by the effects upon the respiratory and cardiac functions, which are intimately related with those of the medulla and cord.

*c.* With regard to the influence of acetanilide upon the peripheral nervous system, we have already observed that sensation is diminished especially in the posterior part of the body, where it may reach a state of complete anæsthesia. Besides these there are vaso-motor disturbances, which are particularly noticeable in the region of the ear. The vaso-motor disturbances are produced indirectly by the action of the drug upon centers located in the medulla oblongata, the region where the heat center is also found.

§ 3. *Mechanism of the Action of Acetanilide.*—If we now attempt with the data which have just been set forth to give an explanation of the physiological action of the medicine, we may safely maintain the following :

1. The drug has a definite action upon the elements of the blood.
2. It also has a marked effect upon the nervous system.

To these two factors we must attribute the action of acetanilide, although we cannot state whether these modes of action are entirely distinct or interdependent. It is certain, however, that blood changes are effected and the nervous symptoms may either result secondarily from these or the drug may act primarily upon the nerve centers. In either case the activity of the phenomena of nutrition are reduced, as manifested by the slowing of the heart and respiration, the reduction of temperature, the diminution of sensation, etc. When however we transport our investigations to the domain of pathology and endeavor to harmonize the data which we have obtained from physiological researches, experiments which are always under the control of the investigator, with the varying conditions found in disease, we are compelled to believe that the most marked effect of the drug is manifested by the changes produced in the nervous system. Indeed, in many patients treated by acetanilide the action of the drug seemed to be exclusively upon the nervous system. How else can we explain the anæsthetic action observed without any effect upon the temperature, or any of the disturbances which follow important changes in the blood? It would seem that in medicinal doses acetanilide affects only the exaggerated and abnormal action of the centers located in the bulb and cord. Toxic doses on the contrary seem to attack at once the normal physiological fountains of the bulb and at the same time profoundly alter the character of the blood. In this way we may explain how the drug acts at one time exclusively upon the temperature, at another upon other functional phenomena dependent upon centers located in the myelaxis, the temperature not being affected in the least.

*Therapeutics of Acetanilide.*—We have used acetanilide in fourteen cases in the service of Prof. Dujardin-Beaumetz, at the Hopital Cochin, as follows: Two cases of typhoid fever, three cases of acute articular rheumatism, one case of pneumonia, one locomotor ataxia, one uræmic convulsions, one acute meningitis, one spinal sclerosis, two cases of tuberculosis and two of epilepsy. In cases of tuberculosis doses of four grains produced profuse sweating and the patient seemed to be weakened after the use of the drug. The antipyretic effects were not maintained as long as in other febrile diseases. In the light of these facts we conclude that acetanilide cannot be advantageously employed in tubercular affections.

Neither of the two epileptics has had a fit since we began the use of acetanilide in doses of twenty-four grains a day. On account of the short period in which we have used the drug in these cases—one month—we can give no definite conclusion as to its utility. We think however that it may prove of great value in the treatment of this obstinate affection. We do not care to discuss the value of antithermic medication, a subject which has been much criticised of late. We will simply state that acetanilide has a rapid and intense antithermic action without the inconvenience of most other antipyretics. The administration is very simple, it is almost tasteless, it never produces nausea, nor that state of semi-intoxication caused by antipyrin. The cyanosis occasionally observed is accompanied by no alarming symptoms and rapidly disappears.

The amount of acetanilide which will produce an effect equivalent to fifteen grains of antipyrin varies. According to Cahn it is four grains, according to Krieger, six grains, and according to Mouisset, eight grains. This matter requires a comparative study of the two substances, a subject which has not yet been undertaken. In typhoid fever, however, four grains is an amply sufficient dose. Patients with this disease are very susceptible to the drug. In one of our cases a dose of four grains reduced the temperature 3.2°C. (5.8°F.) In another case eight grains caused cyanosis in a robust typhoid patient in the beginning of his disease.

In acute rheumatism the antithermic effects of acetanilide is much less; eight grains may be given twice in twenty four hours. The drug has the double advantage of reducing temperature and pain. We have not had an opportunity to try acetanilide in cerebral rheumatism where the drug would probably prove of great value. As a nervine, acetanilide is indicated in a great number of nervous affections, and seems to be an improvement upon bromide of potassium, as it will act in cases where the bromides are inert. Two of our patients who had "lightning pains" were relieved after many other remedies had been tried in vain.

If on the one hand a tolerance for acetanilide is easily established after prolonged administration, we nevertheless avoid the untoward effects of bromide of potassium, fetid breath, malaise, intellectual hebetude, muscular weakness, eruptions, etc. In nervous diseases acetanilide produces absolutely no effect upon the temperature, and very seldom causes sweating, the digestive functions are undisturbed, nor have we observed any other trouble. In nervous diseases the dose must be larger than in febrile affections. Begin with fifteen grains in twenty-four hours, to be rapidly increased to thirty grains.

In conclusion we will briefly recapitulate the facts ascertained as the result of our investigations :

1. Physiological action.

Acetanilide has a marked effect upon the nervous system manifested.

(a.) By symptoms of collapse after a short period of excitement.

(b.) By general anæsthesia and analgesia.

(c.) By modification of the functions of the heart and blood vessels, as shown by the increased intravascular pressure and peripheral vaso-constriction.

(d.) Central and peripheral reduction of temperature.

(e.) In toxic doses acetanilide profoundly modifies the elements of the blood, particularly the oxyhæmoglobin which is at first reduced and then changed into methæmoglobin. From this results a diminution of the respiratory powers, symptoms of asphyxia and death.

(f.) The mechanism of the action of the drug seems to be principally due to its influence upon the bulbo-medullary cells.

2. Therapy.

(a.) Acetanilide is a powerful antithermic and a valuable nervine.

(b.) As an antithermic it is of the greatest value in all diseases where it is desirable to combat hyperpyrexia.

(c.) As a nervine it is indicated in all nervous diseases characterized by morbid hyper-excitability, such as epilepsy.

(d.) A toleration for the drug is established by its prolonged and uninterrupted administration.

(e.) Diuresis is often diminished, sometimes stationary, but never increased by acetanilide.

